# Involvement of Vasopressin in the Pathogenesis of Pulmonary Tuberculosis: A New Therapeutic Target?

**DOI:** 10.3389/fendo.2019.00351

**Published:** 2019-06-06

**Authors:** Mario Zetter, Jorge Barrios-Payán, Dulce Mata-Espinosa, Brenda Marquina-Castillo, Andrés Quintanar-Stephano, Rogelio Hernández-Pando

**Affiliations:** ^1^Experimental Pathology Section, Department of Pathology, Instituto Nacional de Ciencias Médicas y Nutrición Salvador Zubirán, Mexico City, Mexico; ^2^Departamento de Fisiología y Farmacología, Centro de Ciencias Básicas, Universidad Autónoma de Aguascalientes, Aguascalientes, Mexico

**Keywords:** vasopressin, lung, tuberculosis, immunopathology, fibrosis, therapy

## Abstract

Tuberculosis (TB) is a highly complex infectious disease caused by the intracellular pathogen *Mycobacterium tuberculosis* (Mtb). It is characterized by chronic granulomatous inflammation of the lung and systemic immune–neuroendocrine responses that have been associated with pathophysiology and disease outcome. Vasopressin (VP), a neurohypophysial hormone with immunomodulatory effects, is abnormally high in plasma of some patients with pulmonary TB, and is apparently produced ectopically. In this study, a BALB/c mouse model of progressive pulmonary TB was used to determine whether VP may play a role in TB pathophysiology. Our results show that VP gene is expressed in the lung since early infection, increasing as the infection progressed, and localized mainly in macrophages, which are key cells in mycobacterial elimination. Pharmacologic manipulation using agonist and antagonist compounds showed that high and sustained stimulation of VPR resulted in increased bacillary burdens and fibrosis at lungs, while blockade of VP receptors reduced bacterial loads. Accordingly, treatment of infected alveolar macrophages with VP in cell cultures resulted in high numbers of intracellular Mtb and impaired cytokine production. Thus, we show that VP is ectopically produced in the tuberculous lungs, with macrophages being its most possible target cell. Further, it seems that chronic vasopressinergic stimulation during active late disease causes anti-inflammatory and tissue reparative effects, which could be deleterious while its pharmacologic suppression reactivates protective immunity and contributes to shorten conventional chemotherapy, which could be a new possible form of immune-endocrine therapy.

## Introduction

Tuberculosis (TB) is the leading cause of death by a single infectious agent worldwide (1). It is caused by the intracellular bacillus *Mycobacterium tuberculosis* (Mtb) that affects the lungs mainly and is characterized by chronic and excessive inflammation, in which innate and adaptive immune responses are profoundly affected (2, 3). Infection starts though inhalation of saliva droplets with mycobacteria that reaches alveoli and is then engulfed by alveolar macrophages. Macrophages are key cells in bacilli elimination through different mechanisms (3). Nevertheless, Mtb has evolved several mechanisms to avoid immune responses, and eventually, phagocytic cells become incapable of bacilli clearance (4). Mycobacterial antigens are then processed by dendritic cells and presented to T lymphocytes in regional lymph nodes, and so, a type IV (delayed) hypersensitivity response is generated. Lymphocytes migrate to the lung and, together with fibroblasts, surround infected macrophages and form containment structures known as granulomas, which are the histopathological hallmark of TB (5). Thus, complex interactions between bacterium and host cells occur, determining the outcome of infection. In early stages of active infection, Th1 cellular immune responses are protective, as interferon gamma (IFNγ) and interleukin 12 (IL-12) induce macrophage activation, allowing bacterial growth control; nevertheless, during late active disease, extensive inflammation leads to a shift toward a Th-2 immune response in which IL-4, IL-10, and transforming growth factor-β (TGF-β) induce a local anti-inflammatory and immunosuppressive milieu resulting in poor containment of infection and progression of tissue damage, necrosis, and fibrosis, driving host to death (6). Besides these immunologic features, an intense neuroendocrine response during pulmonary mycobacterial infection creates a complex network of cytokines, hormones, and neurotransmitters that contribute to the outcome of TB pathogenesis (7, 8).

During pulmonary TB, different hormonal and neuroendocrine pathways are dysregulated, modifying the immune response to Mtb and influencing the outcome of infection. Neuroendocrine dysfunction and hormonal resistance have been found during human and experimental pulmonary TB (9). Further, the hypothalamus–pituitary–adrenal (HPA) axis seems to be chronically activated, a situation that worsens immunopathology, allowing disease progression (10). The hypothalamus is indeed a central anatomical area in which neuroimmune responses are integrated. Frequently, it is also affected by peripheral inflammation, and noteworthy, after intense inflammatory stress such as caused by Mtb.

The inappropriate production of VP during TB has been extensively reported; in fact, evidence of altered water metabolism was observed more than half a century ago (11). Furthermore, an “antidiuretic principle” was found in lungs of patients with active pulmonary TB that appears to be independent from the hypothalamus (12, 13), suggesting a direct involvement of the vasopressinergic system (VS) in the pathophysiology of TB.

Vasopressin (VP) is a well-evolutionary-conserved cyclic peptide conformed by nine amino acid residues, produced physiologically in parvocellular and magnocellular neurons in the paraventricular and supraoptic nucleus of the hypothalamus. It is synthetized as a long precursor molecule (Neurophysin II–VP–copeptin) (14), which is cleaved by endoproteases and released to median eminence and general circulatory system as a response of different central and peripheral stressors including hypovolemia, hyperosmolarity, and dehydration (15–18). Furthermore, it has been shown that a VP is released as a response to peripheral inflammation (19, 20) and that this response could result deleterious in different immune-mediated diseases. VP exerts biological effects via at least three G-protein-coupled receptors named V1a, V1b, and V2, which are ubiquitously distributed (21).

Immune modulatory effects of VP are required early during monocyte/lymphoid ontogeny, and it is necessary to homeostatic lymphoid and myeloid development, as seemed in VP-deficient rats (Brattleboro strain), which present subtle but basal immunodeficiency, particularly in macrophage function (22, 23). In the context of inflammatory challenges, vasopressinergic activity is required during early and late stages, as VP coactivates the HPA axis among corticotrophin-releasing factors (CRFs), inducing cortisol production (24, 25). Conversely, in chronic inflammation, VP appears to be the main cortisol secretagogue (26, 27). Besides, in endothelial cells, VP regulates the expression of chemokines responsible for leukocyte migration and modulates the production of inflammatory cytokines by fibroblasts and macrophages (28–30). Adaptive immunity is also influenced by VP as it replaces IL-2 requirement of T lymphocytes for cytokine production and acts like a mitogen (31–33). Basal vasopressinergic *tone* is required for antibody production while it down-regulates the expression of B cell receptor (34, 35). Immunomodulatory effects of VP are dose and time dependent and differ between organs and tissues. In the urinary tract, vasopressinergic activity results in an epithelial milieu that impairs immune response against pathogenic bacteria (36). Further, in lungs, it has been reported that VP inhibits the translocation of nuclear factor kappa B (NF-κB), resulting in a decreased IL-6 production and reduced pulmonary inflammation in response to lipopolysaccharide (LPS) (37). VP is a pleiotropic molecule that participates in the maintenance of homeostasis, but also seems to contribute to the establishment of certain diseases characterized by excessive inflammation and tissue remodeling such as cancer and autoimmunity and probably in chronic infections like TB. Nevertheless, this last point has not been studied in detail.

Thus, the aim of this study was to determine the role of the VS in Mtb infection. Using a model of progressive pulmonary TB in BALB/c mice, the kinetics of gene expression and production of VP in the lungs during mycobacterial infection was determined. To study the VP contribution in the pathogenesis of the disease, infected mice were treated with VP agonist and antagonist during the early and late phase of the disease and cell culture bacterial killing assays were made.

## Materials and Methods

### Ethics Statements

All the animal work was done according to the guidelines of the Mexican law NOM 061-Z00-1999 and approved by the Internal Committee for the Care and Use of Laboratory Animals (CICUAL) of the National Institute of Medical Sciences and Nutrition in México (Protocol number PAT-1861-16/20).

### Experimental Model of Progressive Pulmonary TB in BALB/c Mice

The experimental model of progressive pulmonary Tb has been described previously (6). Briefly, the *M. tuberculosis* reference strain H37Rv (ATCC No. 25618) was grown in Middlebrook 7H9 broth (DIFCO) supplemented with 0.2% glycerol, 10% OADC enrichment, and 0.02% Tween-80 and maintained at 37°C in agitation. Mid log-phase cultures were used for all the experiments. Mycobacteria were counted and stored at −80°C until use. Bacterial aliquots were thawed and pulse-sonicated to remove clumps. For the infection, male BALB/c mice (*n* = 36), 8 weeks old and weighing 21–23 g, were anesthetized in gas chamber using Sevofluorane and infected intratracheally with 2.5 × 10^5^ live bacilli using a cannula inside a biosafety level III cabinet. Mice were maintained in vertical position until spontaneous recovery and maintained in groups of five in cages fitted with microisolators connected to negative pressure in animal biosafety level III facilities. Groups of five animals were euthanized inside a cabinet of biosecurity level III at 1, 3, 7, 14, 21, 28, and 60 days post-infection by exsanguination under anesthesia with 210 mg/kg of intraperitoneal pentobarbital. Three left lungs per time were perfused with absolute ethanol, fixed, and prepared for histopathological studies. After eliminating hilar lymph nodes and thymic tissues, seven lungs were frozen and kept to −80°C for bacilli loads determination and gene expression studies in two separated experiments. Animals were monitored daily and humanely euthanized under pentobarbital anesthesia if respiratory insufficiency, accentuated cachexia, or total immobilization was noted.

### Preparation of Lung Tissue for Morphological and Immunohistochemical Analysis

Lungs of infected mice were perfused with absolute ethanol by endotracheal route and fixed for 24 h and then embedded in paraffin blocks. Sections of 4 μm were obtained with a microtome, mounted on glass slides, deparaffinized, and stained with hematoxylin and eosin or with Masson's trichrome staining method. For quantification and morphometric analysis, three different mouse lungs per time point were evaluated. Pneumonic areas were measured with a histology automated system (Leica Microsystems), and then the percentage of affected area in microns was reported from two different experiments. For immunohistochemistry, lung tissues were sectioned and mounted on glass slides and then deparaffinized. Slides were first blocked for unspecific activity of peroxidase with methanol peroxide 3% for 1 h. For VP tissue detection, a rabbit anti-mouse polyclonal antibody (Genetex, USA) was used at a concentration of 1:100, incubated overnight in agitation, followed by incubation with secondary anti-rabbit IgG labeled with peroxidase. For TGF-β immunostaining, a rabbit anti-mouse polyclonal antibody directed to TGF-β1 isoform was used at a final concentration of 1:250. In both cases, bound antibodies were detected with diaminobenzidine and counterstained with hematoxylin.

### Gene Expression Kinetics of VP, VPR, and TGF-β in Lung Homogenates

The right lungs of three of the euthanized mice were obtained and stored in 1.5-ml cryotubes, immediately frozen in liquid nitrogen, and maintained at −80°C until processing. For homogenization, lungs were slowly defrosted and zirconium flint beads were added to each tube and lung tissue was homogenized in the FastPrepR-24 (MP Biomedicals). RNA extraction was performed with the RNeasyR Mini kit (Qiagen) following the manufacturer's instructions. The RNA obtained was quantified by spectrophotometry (A260/280), 100 ng of RNA from lung was used for the production of cDNA by retro-transcription following the indications of the Omniscript kit (Qiagen), and later an endpoint PCR was run to amplify the constitutive gene RPLP0 (Ribosomal protein large P0, Gen ID:11837, GenBank, NCBI) and its integrity was analyzed by running at 2% agarose gel stained with SYBR green. Complementary DNA (cDNA) obtained from each sample was analyzed by real-time PCR (qPCR) using the Real-Time PCR system 7500 (Applied Biosystems) and the Quantitech SYBR Green Mastermix kit (Qiagen) with specific primers (Invitrogen) designed with the first-BLAST (ncbi.nlm.nih.gov) for VP, V1aR, and V2R. For absolute quantification, the number of copies of each target gene was normalized to 1 million amplicons of mRNA of the housekeeping gene RPLP0, including the standard curves and a negative control. Cycling conditions used were as follows: initial denaturation at 95°C for 15 min, followed by 40 cycles at 95°C for 20 s, 60°C for 20 s, and 72°C for 34 s. In the case of TGF-β, for relative quantification, the Ct values were determined by 7500 real-time PCR system (Applied Biosystems, Foster City, CA, USA), and the fold change of gene expression was calculated by the 2^−(△△Ct)^ method (38). Sequences of primer probes can be found in Supplementary Table 1.

### Pharmacological Manipulation of Vasopressinergic System

For the pharmacological treatment during early infection, male mice (*n* = 60) were infected as mentioned above and divided into two different groups: (1) saline control group (SS, *n* = 30) and (2) desmopressin (DdAVP, *n* = 30). Treatments were administered twice a day from day 1 post-infection and during the first 2 months. Animals were euthanized in groups of five in each time point (days 3, 7, 14, 21, 28, and 60). Three lungs were perfused and embedded in paraffin, and seven lungs were frozen immediately and stored at −80°C until processing for CFU counting as mentioned above and used for PCR analysis. In another set of experiments (late disease treatment), infected mice (*n* = 120) were divided into four groups: (1) saline control group (SS, *n* = 30), (2) desmopressin (DdAVP, *n* = 30), (3) conivaptan (CVP, *n* = 30), and (4) control vehicle (DMSO 10%) group (*n* = 30). Treatments were administered twice a day for 2 months, starting on day 60 of infection. Groups of 5 mice were euthanized on days 75, 90, and 120 post-infection (days 15, 30, and 60 of treatment). It is important to mention that DdAVP is a selective agonist of V2 receptor with weak affinity for V1a and V1b receptors, while CVP is a non-peptidic antagonist of V1aR/V2R with weak effects on V1b receptor. Two independent experiments were performed. The dose of DdAVP was 0.75 μg/kg twice a day in a volume of 10 μl (vehicle was 0.9% NaCl solution) administered intramuscularly, which is the necessary dose to restore antidiuresis in neurointermediate-lobectomized animals (39). CVP obtained from BiochemPartner (BCP07817, Shangai) was diluted in sterile water for injection with DMSO [10%] and administered via intramuscular (1 mg/kg twice a day in a final volume of 10 μl).

As shown later in the Results section, the group of infected mice treated with CVP during the advanced phase of the disease showed a significant decrease in the lung bacillary load, which suggests that the administration of this VP receptor blocker could have synergistic effect when administered in conjunction with conventional antibiotics used in the treatment of TB, with the objective of shortening the treatment. To study this aspect, mice (*n* = 120) on the 60th day of infection were divided into four groups (*n* = 15, each): the first group was a vehicle control (DMSO 10%); the second group received CVP at the same dose mentioned above; a third group (AB) was treated with first-line antibiotics, which are isoniazid (10 mg/kg of weight), rifampicin (10 mg/kg of weight), and pyrazinamide (30 mg/kg of weight), administered every day with an intragastric cannula in a volume of 100 μl; and a fourth group of antibiotic plus conivaptan (AB + CVP, *n* = 15) received the same type of antibiotic therapy plus CVP intramuscularly (1 mg/kg twice a day). All experimental groups were treated for 2 months. Groups of five animals were euthanized on days 15, 30, and 60 post-treatment. The right lungs were used to determine bacillary load and the left lungs were used to analyze the extent of the pneumonic damage by automated morphometry.

### Collagen Quantification by Hydroxyproline Assay

To determine the extent of fibrosis during the advanced phase of pulmonary Tb, the amount of hydroxyproline was determined as an indirect measure of the amount of collagen in lung expressed in milligrams per gram of dry tissue. All reagents were obtained from the Hydroxyproline Quantification Kit (Sigma-Aldrich). Briefly, the right lungs of three mice per group treated with DdAVP or controls were dehydrated at 60°C and hydrolyzed with 1 ml of HCl (6N) and incubated at 110°C overnight. Subsequently, the samples were neutralized with NaOH (pH 7) and filtered and 50 μl of sample was diluted in 2 ml of distilled water. Chloramine T was added to each tube (1 ml), mixed, and incubated for 25 min at room temperature. Then, 1 ml of perchloric acid (3.15 M) was added and incubated for 5 min at room temperature. Finally, 1 ml of *p*-dimethylaminobenzaldehyde and ethylene glycol was added. The tubes were placed in a water bath at 60°C for 20 min and then cooled in water for 5 min. Samples were placed and a standard curve was made from a hydroxyproline standard of 10 μg/ml. Each sample was analyzed in triplicate. The absorbance was read at 557 nm in a spectrophotometer. To determine the collagen concentration, the values obtained from hydroxyproline were multiplied by the dilution factor and then by the constant 7.23.

### Mycobacterial Killing Assay in Cell Cultures

Murine Balb/c alveolar macrophages (cell line MH-S, ATCC® CRL-2019) were seeded in 96-well plates (1 × 10^4^ cells per well) in RPMI medium (Caisson Labs, USA, Cat. RPL03) supplemented with 5% fetal bovine serum (Gibco, USA) and pre-stimulated and treated with three different doses: low (10^−8^ M), medium (10^−7^ M), and high (10^−6^ M) doses of DdAVP (Merck, Ger) or CVP (10^−6^ M) for 12 h. The dose of CVP corresponded to the molar higher dose of DdAVP making an equimolar inhibition. Subsequently, cells were infected with Mtb strain H37Rv at an MOI of 1:3 for 1 h. The wells were then washed with RPMI/Amikacin medium to eliminate non-phagocytosed bacteria. Infected cells in wells corresponding to 1 h post-infection time were lysed with 1% SDS and incubated for 10 min, and then 20% bovine serum albumin (BSA) was added to stop reaction. Serial dilutions from an initial volume of 10 μl were made in culture broth (7H9, Middlebrook, USA) and seeded in solid culture medium (7H10). The number of live bacteria was determined by counting colony-forming units (CFUs) as previously described (40). In the 24-h wells, the infected macrophages were supplemented every 12 h with different doses of DdAVP or CVP, and at the end of the experiment, the same procedure of preparation and sowing of bacteria in 7H10 medium was performed. A second experiment was performed in order to study the effects of sustained vasopressinergic stimulus on infected macrophages. 1 × 10^5^ MH-S cells were seeded and infected as mentioned above in 12-well plates, with 1 ml of RPMI medium, and treated with the highest dose of VP and CVP. Culture medium (RPMI) was daily supplemented with VP or CVP at 9:00 h and followed in a kinetic of 72 h. Cells were harvested as described above for CFU count and culture supernatants were collected in 1.5-ml tubes containing 50 μl of protease inhibitor for cytokine detection.

### Cytokine Detection

Cytokines IL-6 and TNFα were measured in pools of culture supernatants from control-infected treatment groups and non-infected macrophages employing commercially available ELISA kits according to the manufacturer's instructions (BioLegend Company, CA, USA). Detection limits were 63.0–4,000 pg/ml for IL-6 and 15.6–1,000 pg/ml for TNFα.

### Mycobacterial Culture Assays

In order to test the possibility of the effect of AVP directly on mycobacteria, 1 × 10^5^ CFU of *M. tuberculosis* (H37Rv) were seeded on 96-well plates in 200 μl of liquid broth media supplemented with the higher (1 × 10^6^ M) and lower (1 × 10^10^ M) dose of synthetic AVP (Sigma-Aldrich, Germany) or DdAVP in groups of three for each experimental condition, and incubated in soft helicoidal agitation at 37°C in a CO_2_ (3%) atmosphere for a 7-day period. At the end of this experiment, 40 μl of [3-(4,5-dimethylthiazol-2-yl)-5-(3-carboxymethoxyphenyl)-2-(4-sulfophenyl)-2H-tetrazolium, inner salt; MTS] (Cell-titer 96) was added to each experimental well, and the transformation of the resulting formazan was read 4 h before. This compound is turned enzymatically to formazan by electron transport chain of viable mycobacteria (41). Before the MTS assay, 10 μl was seeded in solid (7H10) medium and counted on day 21 as mentioned above.

### Statistical Analysis

All the statistical analysis was performed using GraphPad Prism Software (version 6.0, La Jolla, USA). The data were analyzed using paired *t*-test, one- and two-tailed ANOVA with Bonferroni correction for multiple comparisons. *P-*values < 0.05 were considered significant.

## Results

### Local Vasopressinergic Activity During Pulmonary Tb

In our TB murine model, there are two phases, an early phase of approximately 21 days in which the Th1-type response in the lung increases progressively and is predominant at the end of this stage, and the progressive phase that starts at day 28 post-infection in which the Th1 cytokine pattern decreases and the Th2-type response emerges in co-existence with extensive inflammatory infiltrate and progressive pneumonia, as well as an increase in the bacillary loads, which leads the animal to death (6).

**Figure 1:**
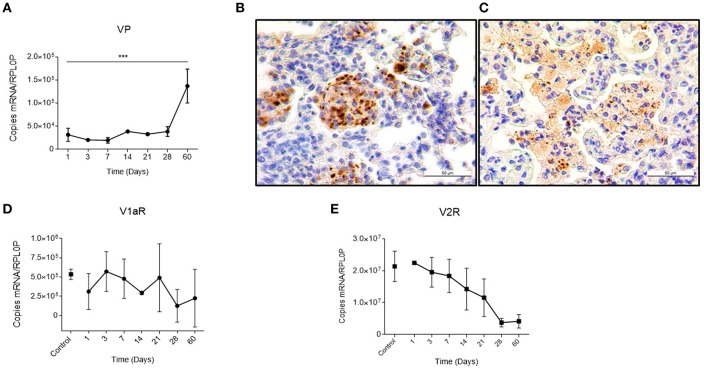
Local kinetics of VP expression and VPR during pulmonary tuberculosis **(A)** Absolute expression of VP mRNA in lung homogenates during the course of infection. **(B)** Representative micrographs of immunohistochemistry with anti-VP antibody of the infected lung on day 14 showing immunopositive macrophages forming a granuloma (400×). As well as in foamy macrophages of pneumonic areas on day 60 post-infection **(C)**. **(D,E)** Absolute quantification of V1a and V2 mRNA, respectively, in lungs of healthy (control) and infected mice. Data are expressed as means ± SD of three different animals at each point. Asterisks represent statistical significance (^***^P < 0.0001, one-tailed ANOVA).

Previously, it was reported that during active TB, there is a compound in the lung that has the same antidiuretic activity as VP, which suggested a possible ectopic production (12, 13). We studied this in our pulmonary TB model. In mice, during the early phase of the infection, there is a gradual granuloma formation since day 14 that coincides with a progressive increase in VP gene expression in the lungs (Figure 1A). Noteworthy, no VP mRNA was found at the healthy lung. These results well-coincided with an increase in the VP positivity detected by immunohistochemistry on the surface of some pneumocytes and strongly positive in macrophages that were forming part of the granulomas (Figure 1B). As the infection progressed, in the pneumonic areas, numerous macrophages with extensive cytoplasm vacuolization (foamy cells) exhibited strong VP immunostaining (Figure 1C) in co-existence with the high number of VP transcripts and progressive decrease of both VP receptors determined by qPCR that was pronounced in V2R (Figures 1D,E).

### Effects of Vasopressinergic Pharmacological Manipulation in the Course of the TB

**Figure 2:**
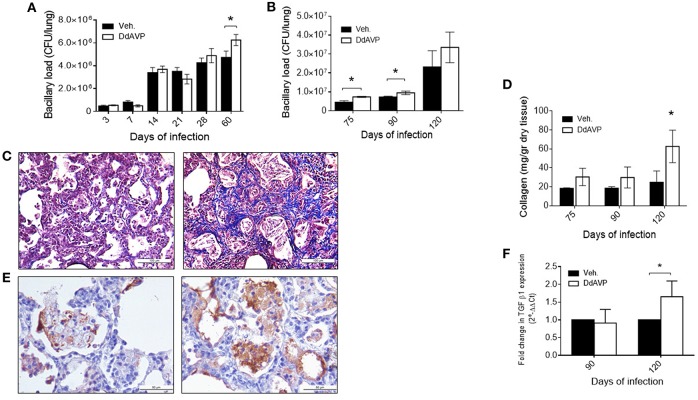
Effect of vasopressin agonism during early and late infection in bacilli loads and histopathology **(A)** Bacillary loads of lung homogenates of mice infected with *M. tuberculosis* strain H37Rv and treated with DdAVP twice a day during early infection. **(B)** Bacillary loads at lungs of mice treated during late infection, starting from day 60 onwards. **(C)** Representative Masson's trichrome stain micrographs of lung of control-infected (left) and DdAVP-treated infected mice (right) on day 120 post-infection. **(D)** Collagen amount in lungs of infected mice treated during the late infection quantified by hydroxyproline assay. **(E)** Representative micrographs of lung immunohistochemistry anti-TGF-β1 of control-infected (left) and DdAVP-infected (right) mice after 60 days of treatment. **(F)** Relative expression of TGF-β mRNA in lungs of infected mice treated with DdAVP during the late infection. Days 75, 90, and 120 of infection correspond to 15, 30, and 60 days of treatment, respectively. Data are expressed as means ± SD of three different animals at each point. Asterisk represent statistical significance (^*^P < 0.05, two-way ANOVA).

Due to the above results and the observations described previously on the immunomodulatory effects of VP, we decided to study its possible effects on experimental TB by pharmacological manipulation. To do this, groups of infected mice were treated with the synthetic agonist desmopressin (8-deamino-arginine VP, DdAVP) or the non-peptide antagonist conivaptan hydrochloride (CVP). DdAVP is a potent agonist of the V2 receptors and has a prolonged half-life in comparison with VP. Treatment with DdAVP during the first month, during the early phase of murine TB, show no effects on histopathology; however, at the end of the infection, a higher number of bacilli was observed in the lungs of these mice in comparison with the controls (Figure 2A). This prompted us to study VP effects during the late infection. In the group of mice treated during the progressive phase of the disease, when the Th2 response and the anti-inflammatory and repair phenomena were predominant (from day 60), a significant increase in the bacillary loads (Figure 2B) and histologically extensive areas of fibrosis in the pulmonary interstitium was observed in mice treated with DdAVP (Figure 2C), which correlated with higher amount of collagen (hydroxyproline) (Figure 2D).

Regarding the possible mechanism of the highly fibrotic response produced by DdAVP administration, we considered the possibility that this could be induced indirectly by increased production of TGF-β, a potent fibrogenic and immunosuppressing cytokine related to poor protection against infection. It has been previously reported that VP promotes collagen synthesis and proliferation of fibroblasts through TGF-β, modifying fibrotic responses (42, 43). In this regard, mice treated with DdAVP showed higher TGF-β immunostaining after 60 days of treatment (Figure 2E, images), which correlated with relative increase in its gene expression (Figure 2F). Thus, it is possible that in advanced TB, VP promotes anti-inflammatory and fibrotic repair by the induction of TGF-β production.

**Figure 3:**
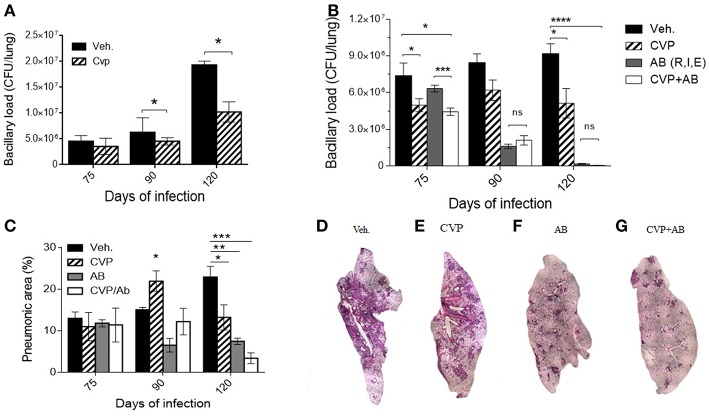
Blockade of VP receptors decreased bacillary loads at lung. **(A)** Comparison of bacillary loads at lung homogenates of control mice treated with vehicle (black-filled bars) and treated with CVP (lined bars) from day 60. **(B)** Effect on bacillary loads of vehicle treated (black-filled bars), CVP (lined bars), the three antibiotics (AB, Rifampicin, Isoniazide, and Ethambutol, gray filled bars) and antibiotics plus CVP (AB + CVP, white bars), during late infection, from day 60. **(C)** Pneumonic areas of the infected lungs of mice treated with the different experimental conditions. **(D–G)** Representative micrographs of automatized reconstruction of infected lungs from vehicle, CVP, AB, and CVP plus AB, respectively. Days 75, 90, and 120 of infection correspond to 15, 30, and 60 days of treatment, respectively. Data are expressed as means ± SD of three different animals at each point. Asterisk represent statistical significance (^*^P < 0.05, two-way ANOVA).

In contrast, in animals treated during the advanced phase of the disease with the competitive non-selective VP antagonist CVP, when the effects of VP agonism were more pronounced, a significant reduction of the bacillary loads was observed (Figure 3A). These results prompted us to test a possible synergistic effect of CVP with conventional antibiotics (rifampin, isoniazid, and ethambutol) on animals in the chronic phase of H37Rv mycobacterial infection.

Although TB can be cured with these antibiotics, the treatment extend at least half a year, which frequently provokes therapeutic abandoning, causing relapse and emergence of drug-resistant bacteria. Therefore, it is necessary to shorten the treatment, and one possibility is the combined treatment antibiotics plus an immune-regulatory agent, such as in this case, CVP, blocking V1a and V2R activity. Mice on the 60th day of infection were treated daily with first-line antibiotics (AB) or AB plus CVP for 2 months. In comparison with animals treated only with antibiotics, mice that received the combined treatment showed a significant decrease in bacillary load at the start of treatment (2 weeks) (Figure 3B). Furthermore, reduced pneumonic areas were noted when animals were synergistically treated with AB plus CVP (Figures 3C–G), suggesting that this treatment may shorten the time of antibiotic therapy and improve clinical manifestations.

### VP Inhibits Mycobacterial Clearance by Alveolar Macrophages

**Figure 4:**
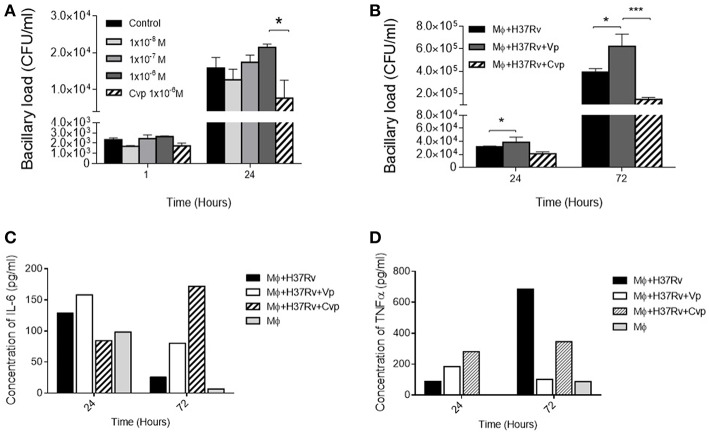
Effects of VP and antagonist CVP on infected alveolar macrophages. **(A)** Number of cultivable intracellular bacilli expressed as CFU per milliliter at 1 and 24 h post-infection in RPMI controls (black bars), incubated with three different doses of DdAVP (1 × 10^−8^ M, white bars; 1 × 10^−7^ M gray bars, and 1 × 10^−6^ M dark gray bars) or CVP (1 × 10^−6^ M, lined bars). **(B)** Effect of VP or CVP on mycobacterial killing by MHS cells at 24 and 72 h post-infection. **(C)** Quantification of IL-6 and TNFα **(D)** in pools of supernatants of infected MHS cells detected by ELISA. Data are expressed as means ± SEM of three different wells at each point. Asterisk represent statistical significance (^*^P < 0.05, two-way ANOVA).

The highest VP immunostaining was exhibited by macrophages; hence, the cell line MH-S of murine alveolar macrophages was the most convenient for the *in vitro* experiments, infecting adherent cells with Mtb and then three different doses of the synthetic agonist DdAVP or the antagonist CVP were added to RPMI medium. After 24 h of infection, repeated administration (at 0, 12, and 24 h) of the highest concentration of DdAVP produced an increase in the number of intracellular bacilli (Figure 4A) in a dose- and time-dependent manner, while CVP produced a significant decrease of bacillary loads when compared with the highest dose of DdAVP, suggesting that a *vasopressinergic tone* modifies the macrophage ability to kill mycobacteria. Moreover, infected macrophages supplemented with AVP (1 × 10^−6^ M) each for 24 h showed higher intracellular bacilli at days 1 and 3 post-infection (Figure 4B), in co-existence with decreased IL-6 in supernatants, which was totally reversed with CVP treatment (Figure 4C). TNFα, a protective cytokine during early mycobacterial infection, was found reduced with AVP treatment; nevertheless, CVP did not reverse the TNF inhibition mediated by VP completely (Figure 4D), suggesting a different mechanism regarding vasopressinergic modulation over macrophage cytokine production. It is important to mention that VP was not synthetized by cultured alveolar macrophages, as they do not express VP mRNA (Supplementary Figure 2).

### Effect of Vasopressin on *M. tuberculosis*

**Figure 5:**
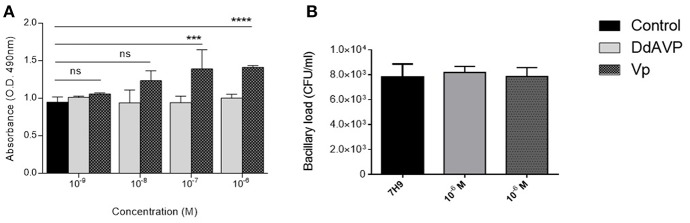
Effects of VP on mycobacterial metabolism. **(A)** Reduction of MTS tetrazolium salts into colored compound formazan by Mtb H37Rv incubated in liquid 7H10 (black bars), medium plus DdAVP (clear gray bars), or medium plus VP (dark gray bars) for 7 days. **(B)** Number of cultivable bacilli in wells of the three experimental conditions. Data are expressed as means ± SD of three wells in two independent experiments. Asterisk represent statistical significance (^***^P < 0.001, ^****^P < 0.0001 two-way ANOVA.

Another aspect of interest was the possibility that VP could be exerting a direct effect on Mtb. To study this, bacteria were seeded in liquid medium in 96-well plates and incubated with lyophilized VP (Sigma-Aldrich) or DdAVP at different concentrations. As mentioned above, DdAVP is an isomer of VP that has an arginine in position D (unlike the position L in VP), which confers, in addition to a prolonged half-life, a greater affinity for the V2 receptor; on the other hand, VP has a similar affinity for its three receptors. After 7 days of incubation, the bacteria that were incubated with VP transformed a greater amount of MTT-tetrazolium into formazan, which did not occur when the bacteria were incubated with DdAVP (Figure 5A) or the medium alone. This bacterial reaction is carried out by a reduction reaction of the compound NADH and is non-reversible, so that the intensity of the positivity indirectly indicates higher metabolic activity (or an increased number of bacteria) in Mtb incubated with VP. Unexpectedly, neither AVP nor DdAVP caused a change in the number of cultivable bacilli in the medium (Figure 5B).

## Discussion

The results shown here are a new example of the constant communication and superposition of neuroendocrine and immune functions during chronic infectious diseases. During ontogeny, the lung epithelium derives from neuroendocrine cells, and during the adult life for its adequate function, airway epithelium requires the influence of hormones and neuropeptides (44, 45). This also contributes to the distinctive characteristics that confirm the lung as an immune-privileged organ, since tolerance to environmental immunogens of the most diverse nature and origin is necessary; however, the lung is also an organ of entry and lodging of many highly evolved microorganisms, such as Mtb. Thus, the lungs need the contribution of well-balanced immune-neuroendocrine response to eliminate diverse infecting agents and efficient cellular mechanisms that repair and restitute the pulmonary tissue after bacterial aggression. In this regard, VP could be a participating factor in the immunopathogenesis of pulmonary TB, considering that it is a pleiotropic hormone that exerts different effects on epithelial, immune, and fibroblast cells, and contributes to the regulation of different phenomena of resistance to inflammatory stress (31, 37, 46). On the other hand, its effects on phagocytosis, cytokine production, and apoptosis also suggest that vasopressinergic dysfunction could contribute to the pathogenesis of several infectious diseases, including TB. No less important is the association that exists between stress-mediated immunosuppression by VP and its relationship with infectious/inflammatory diseases (47–49), as VP is one of the main mediators of the hypothalamic–pituitary–adrenal axis, especially in periods of chronic stress resulting in dysregulated cortisol production (25, 50). In fact, various inflammatory diseases exhibit an increased “vasopressinergic tone”; in particular, lung inflammation of different etiologies is accompanied by this hormonal characteristic (51–55).

In the present work, the existence of the so-called antidiuretic principle in pulmonary TB (13) is confirmed and corresponds to VP, as demonstrated by our immunohistochemical results; the intensity and the number of cells positive for VP in lung increased as the disease progressed, correlating with the extension of pneumonia, and interestingly, foamy macrophages that are the prevalent cell type in the pneumonic areas were those that showed the strongest VP immunostaining, which well-correlated with what was reported, that is, that these types of cells are distinctive in the advanced stage of the disease and are characterized by having a large number of bacteria in their cytoplasm (56). The detection and progressive increase of VP transcripts in infected lungs during infection was seen, suggesting that VP has a significant activity in the infected organ, as there was no VP mRNA expression in the healthy lung.

The effects of VP are highly dependent on its concentration, time of action, type of organ, and target cell. Previously, it has been reported that VP disturbances occur in the pathophysiology of inflammatory diseases (24, 39); nevertheless, the anti-inflammatory effect of this hormone in the lungs has also been described, through the inhibition in the production of IL-6 (37). In addition, in heart, liver, and in fibroblasts, VP is an inducer of fibrosis, a mechanism partially dependent on TGF-β (42, 43, 57). To study the possible pathogenic effect in TB, experiments were carried out using vasopressinergic manipulation, with the synthetic agonist DdAVP, because its half-life is longer than VP, and CVP was used as a non-selective antagonist for the V1a and V2 receptors. The obtained results suggest that VP has anti-inflammatory/profibrotic effects, which should be important to reduce excessive inflammation and to promote the healing of irreversible damaged lung tissue in advanced active TB, which could be modulated by the expression of VP receptors that were lower expressed during progressive late disease, a phenomenon that has been reported previously in response to acute inflammation (58). However, it seems that sustained vasopressinergic activity, such as produced by the administration of DdAVP during late disease, has deleterious effects, as suggested by the higher number of live bacteria and the extensive fibrosis in the lungs of mice treated with this agonist compound. This effect can be mediated through the induction of TGF-β production since immunohistochemistry showed a higher number of strong TGF-β positive foamy macrophages surrounded by areas with extensive fibrosis, and higher gene expression of this cytokine was seen in the animals treated with the VP agonist. TGF-β is an important anti-inflammatory and immunosuppressive cytokine that is produced during active TB and has a significant effect in preventing excessive inflammation, but this activity is produced by inhibiting the TB protective Th1 pro-inflammatory response; hence, TGF-β has a deleterious effect in this disease (59). This cytokine also induces fibroblast activation and collagen secretion, being a key factor in the development of fibrosis. Thus, it seems that Mtb “takes advantage” of the physiological response of VP, which promotes anti-inflammation and tissue healing, leading to a non-protective immunity consequently. These functions can also be important to decrease the pulmonary functional capacity because of fibrosis.

The lower bacillary loads produced by the inhibition of VPR activity in advanced TB mice treated with CVP confirm the possible deleterious effect caused by the continuous and local production of VP on advanced infection and open the possibility of using these observations for therapeutic purposes, specifically the use of VP-blocking agents as adjuvants of antibiotic therapy. In that sense, it is interesting that animals with advanced progressive TB treated with first-line antibiotics and the VP receptor antagonist reduced significantly and more rapidly the bacillary loads in lungs than animals treated only with antibiotics. However, it is important to mention that changes in diuresis and water intake in animals treated with CVP (Supplementary Figure 1) could be interfering with the antibiotic activity; thus, future investigations using selective blockers with no aquaretic activity could be useful. Although TB can be cured by chemotherapy, the treatment usually requires four specific drugs and 6 months of therapy in humans; this long treatment frequently produces significant compliance problems with the consequence of disease recrudescence and the arising of multi-drug resistant (MDR) strains. Thus, the possibility of shortening the conventional treatment is a basic strategy for the control of this disease. Indeed, the frequency of cases of MDR-TB is increasing, and its treatment is even more expensive and demanding, because it requires eight antibiotics administered during a year and a half. We are currently conducting experiments with mice infected with MDR bacteria and treated with second-line antibiotics and CVP, in order to determine if there is synergy and therefore possible shortening in the time of treatment of MDR-TB.

The results in the present study show for the first time the pathogenic importance of VS in experimental pulmonary TB, as far as we know. However, more experiments are needed to define in more detail the contribution of this hormone in the pathogenesis of TB, such as studying whether Mtb produces a VP-like molecule that causes aberrant biological effects that deregulate the mechanisms of the immune system, resulting in favor of the infection as well as the possibility that Mtb could be driving host VP system. Experimental procedures of this work used only male mice, which can be a limiting factor. It will be interesting to know whether female mice develop this vasopressinergic response in lungs, as differences between gender occur in the pathophysiology of TB (60) and also because of the known VP system differences between male and females. Also, it is necessary to study in detail the mechanisms underlying the decrease in bacillary load by blocking its activity *in vivo* or *in vitro*, since they could be caused by a lower rate of phagocytosis, antigen processing, or the inhibition of immunosuppressive effects of VP (mediated by TGF-β). It is also of interest to study if VP, in conjunction with other peptides and neurotransmitters, could be inducing effects directly on Mtb, influencing its virulence, as VP belongs to a huge group of regulatory peptides that have remained in evolution for more than 500 million years, and due to its pleiotropic effects, it is intuitive to think that a complex and well-adapted bacterium could be taking advantage of these host trophic factors. The results shown here with respect to the greater transformation of formazan by mycobacteria when treated with VP suggest this possibility and open discussion about some type of ancestral interaction between VS and Mtb, as this hormone can increase bacterial metabolism. This series of hypothetical possibilities are part of the perspectives of this work.

## Data Availability

The datasets generated for this study are available on request to the corresponding author.

## Ethics Statement

All the animal work was done according to the guidelines of the Mexican law NOM 061-Z00-1999, and approval of the Internal Committee for the Care and Use of Laboratory Animals (CICUAL) of the National Institute of Medical Sciences and Nutrition in México. Protocol number PAT-1861-16/20.

## Author Contributions

MZ, AQ-S, and RH-P contributed to the background work and conceived the experiments. MZ performed, organized, and analyzed the results. DM-E, BM-C, and JB-P contributed to the design and supervised experimental work. AQ-S provided DdAVP and CVP. MZ and RH-P wrote the manuscript. RH-P provided the funds.

### Conflict of Interest Statement

The authors declare that the research was conducted in the absence of any commercial or financial relationships that could be construed as a potential conflict of interest.
