# Supplementary material for: Involvement of Vasopressin in the Pathogenesis of Pulmonary Tuberculosis: A New Therapeutic Target?
Source: Front Endocrinol (Lausanne). 2019 Jun 6;10:351. doi: 10.3389/fendo.2019.00351 (PMC6563385; doi:10.3389/fendo.2019.00351)
Supplement: Supplementary file 1 [file Table_1.pdf]

Supplementary Table 1

**Target gene**	**5´- 3´**	**Product length**
**Vp**	Fw: GCTGCCAGGAGGAGAACTAC<br>Rv: AAAAACCGTCGTGGCACTCG	**141**
**V1aR**	Fw: AGCATGTGTGACCAC CCT AAG<br>Rv: GGCAGGATATCCAAGAACCCT	**104**
**V2R**	Fw: GACCCCCCTTTGTGTTGCTCA<br>Rv: TCAGGAGGGTGTATCCTTCAT	**206**
**TGF-β**	Fw: GCTGATCCCGTTGATTTCCA<br>Rv: GCTGAACCAAGGAGACG	**100**
**RPLP-0**	Fw: CTCTCGCTTTCTGGAGGGTG<br>Rv: ACGCGCTTGTACCCATTGAT	**108**

Table 1. Oligonucleotide sequences
