# Supplementary figures and images for: Involvement of Vasopressin in the Pathogenesis of Pulmonary Tuberculosis: A New Therapeutic Target?
Source: Front Endocrinol (Lausanne). 2019 Jun 6;10:351. doi: 10.3389/fendo.2019.00351 (PMC6563385; doi:10.3389/fendo.2019.00351)

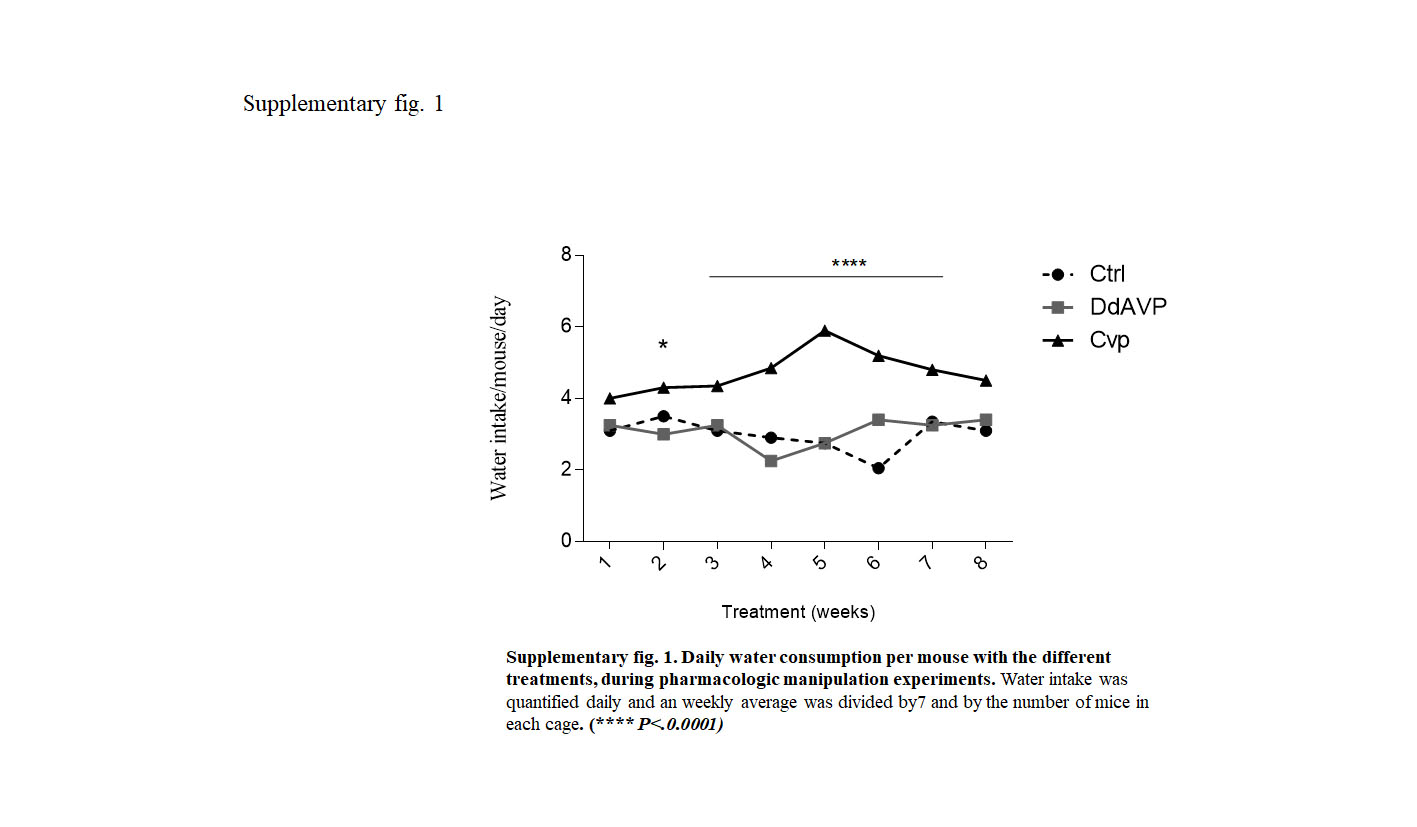

Supplement: Supplementary file 2 [file Image_1.JPEG]

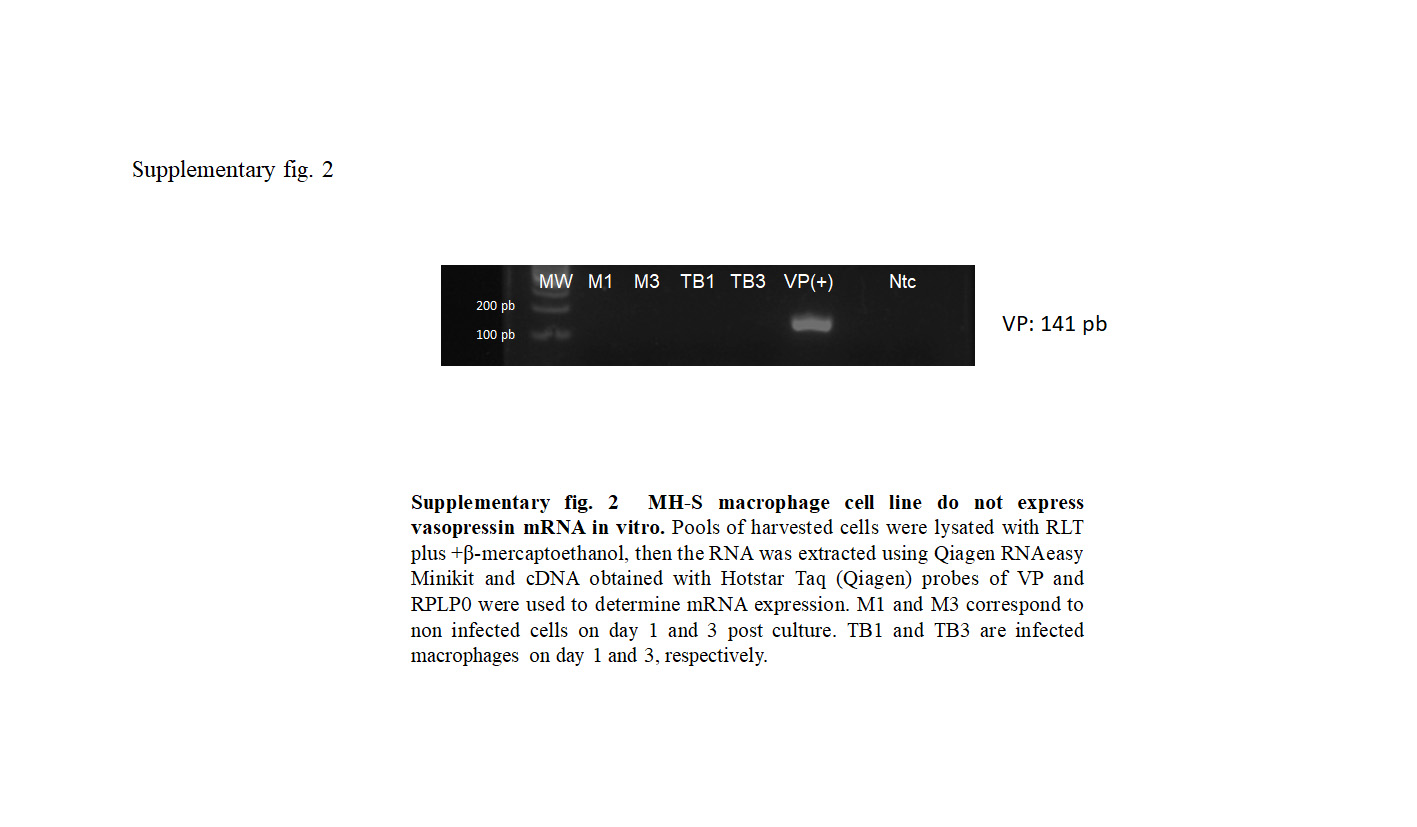

Supplement: Supplementary file 3 [file Image_2.JPEG]
